# Diversification of Quiescin sulfhydryl oxidase in a preserved framework for redox relay

**DOI:** 10.1186/1471-2148-13-70

**Published:** 2013-03-19

**Authors:** Keren Limor-Waisberg, Shifra Ben-Dor, Deborah Fass

**Affiliations:** 1 Department of Structural Biology, Weizmann Institute of Science, Rehovot, 76100, Israel; 2 Biological Services, Weizmann Institute of Science, Rehovot, 76100, Israel

**Keywords:** Disulfide bond formation, Thioredoxin, Flavin adenine dinucleotide, Multi-domain, Protein evolution

## Abstract

**Background:**

The enzyme family Quiescin Sulfhydryl Oxidase (QSOX) is defined by the presence of an amino-terminal thioredoxin-fold (Trx) domain and a carboxy-terminal Erv family sulfhydryl oxidase domain. QSOX enzymes, which generate disulfide bonds and transfer them to substrate proteins, are present in a wide variety of eukaryotic species including metazoans and plants, but are absent from fungi. Plant and animal QSOXs differ in their active-site amino acid sequences and content of non-catalytic domains. The question arises, therefore, whether the Trx-Erv fusion has the same mechanistic significance in all QSOX enzymes, and whether shared features distinguish the functional domains of QSOX from other instances in which these domains occur independently. Through a study of QSOX phylogeny and an analysis of QSOX sequence diversity in light of recently determined three-dimensional structures, we sought insight into the origin and evolution of this multi-domain redox alliance.

**Results:**

An updated collection of QSOX enzymes was used to confirm and refine the differences in domain composition and active-site sequence motif patterns of QSOXs belonging to various eukaryotic phyla. Beyond the expected phylogenetic distinction of animal and plant QSOX enzymes, trees based on individual redox-active QSOX domains show a particular distinction of the Trx domain early in plant evolution. A comparison of QSOX domains with Trx and Erv domains from outside the QSOX family revealed several sequence and structural features that clearly differentiate QSOXs from other enzymes containing either of these domains. Notably, these features, present in QSOXs of various phyla, localize to the interface between the Trx and Erv domains observed in structures of QSOX that model interdomain redox communication.

**Conclusions:**

The infrastructure for interdomain electron relay, previously identified for animal and parasite QSOXs, is found broadly across the QSOX family, including the plant enzymes. We conclude that the conserved three-dimensional framework of the QSOX catalytic domains accommodates lineage-specific differences and paralog diversification in the amino acid residues surrounding the redox-active cysteines. Our findings indicate that QSOX enzymes are characterized not just by the presence of the two defining domain folds but also by features that promote coordinated activity.

## Background

The proper pairing and oxidation of cysteine thiols to disulfides is crucial for the folding of many proteins. Accordingly, specialized enzymes catalyze the formation of disulfide bonds [1]. One such enzyme is the eukaryotic Quiescin Sulfhydryl Oxidase (QSOX). QSOX homologs have been found in protists, plants, and metazoans, but not in fungi [2]. Though disulfide catalysts are conserved components of the endoplasmic reticulum (ER), QSOX is localized outside the ER. In particular, plant QSOX is found in the cell wall [3], and mammalian QSOX is localized to the Golgi apparatus [4,5] or secreted from cells [6]. The importance of oxidative protein folding in the early secretory pathway for production of cell-surface and secreted proteins is well-appreciated, but the role of an additional disulfide catalyst downstream of the ER is poorly understood.

The vastly differing substrate sets potentially available to QSOX enzymes in subcellular or extracellular environments across various taxa raise questions regarding QSOX evolution and whether QSOX enzymes in various species have a common role. In particular, a study of QSOX physiological function (Ilani T, Alon A, Grossman I, Horowitz B, Kartvelishvily E, Cohen SR, Fass D: A secreted disulfide catalyst controls extracellular matrix composition and function, submitted) identified a requirement for metazoan QSOX in assembly of extracellular matrix proteins that are not present in plants. A study of QSOX phylogeny will therefore complement the experimental identification of QSOX substrates to distinguish potential general roles for a post-ER disulfide formation pathway from functions into which the enzyme may have been co-opted on particular branches of the evolutionary tree. Furthermore, comparative analysis of QSOX enzymes will aid the identification of structural features that contribute to QSOX catalysis or differentiate QSOX enzymes from one another. Motivated by increasing interest in the biology of this enzyme family and facilitated by the large number of sequences that have become available in recent years, we present an analysis of QSOX evolution, highlighting universal features *vs.* aspects of the enzyme family that may vary for adaptation to particular functional niches.

**Figure 1:**
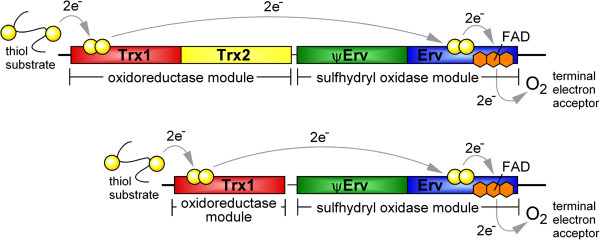
**Schematic representation of the electron transfer pathway through QSOX domains.** As depicted, two electrons are accepted from the substrate by the CXXC motif of the QSOX Trx1 domain, within the oxidoreductase module of QSOX. From the Trx1 domain, the electrons are transferred to the sulfhydryl oxidase module of the QSOX enzyme, first to the CXXC motif of the Erv domain, then to the FAD cofactor. Ultimately, the two electrons are transferred to molecular oxygen, the terminal electron acceptor.

QSOX enzymes comprise an oxidoreductase moiety and a sulfhydryl oxidase moiety [7], which act in tandem to oxidize substrates and transfer the excess electrons to oxygen. This activity occurs through a series of electron-transfer events within the multi-domain protein [8,9] (Figure 1). Such self-sufficiency, *i.e.*, the ability to perform both oxidation of the terminal thiol-containing substrate and reduction of the terminal electron acceptor, distinguishes QSOX from other oxidoreductases and sulfhydryl oxidases that perform comparable reactions sequentially but separately [1]. The oxidoreductase module of QSOX contains a thioredoxin-fold (Trx) domain related to the Protein Disulfide Isomerase (PDI) family and has a redox-active CXXC motif (Trx-CXXC). Trx superfamily domains consist of a central, four-stranded β-sheet between two helices on one side and a single helix on the other. The PDI family is distinguished by an additional disulfide present in a CX_6-8_C motif about 10 Å from the Trx-CXXC motif in the tertiary structure. The sulfhydryl oxidase module of QSOX contains a domain related to the Erv enzyme family. The Erv domain consists of a four-helix bundle and a fifth helix that packs perpendicularly to the bundle; a flavin adenine dinucleotide (FAD) co-factor is bound non-covalently within this helical fold. Here again, a redox-active CXXC motif (Erv-CXXC), situated next to the isoalloxazine of the FAD, serves as the active site.

**Figure 2:**
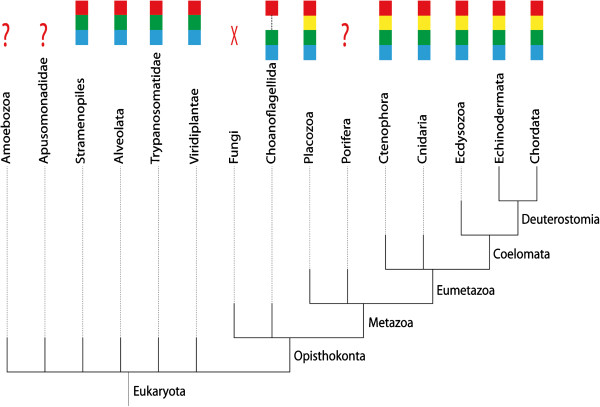
**Domain composition of QSOX in organisms of various phyla.** Domains are colored in accordance with Figure 1: Trx1 (red), Trx2 (yellow), ψErv (green), and Erv (blue). Question marks indicate phyla with limited genomic data available and for which the presence of QSOX has not been demonstrated. An X indicates the presumed absence of QSOX from fungi, for which many genomic sequences are available but no QSOX gene has been detected. A dashed line in the domain map of Choanoflagellida QSOX indicates a lack of sequence information covering this segment.

Between the Trx and Erv domains of QSOX is a helix-rich region revealed by the X-ray crystal structures of mammalian and trypanosomal QSOXs to have a fold similar to that of the Erv domain [9,10]. This region is therefore referred to as a pseudo-Erv domain (ψErv) [10]. Furthermore, in metazoan QSOXs, but not in plant and protist QSOXs (Figure 2), a second Trx-fold domain (Trx2) was identified between the active Trx domain and the ψErv domain [8]. Though they lack catalytic activity, the Trx2 and ψErv domains may influence the dynamics and sterics of the interaction between the redox-active domains in the intramolecular electron-transfer relay, and are thus expected to play an important, albeit indirect, role in catalysis. Another region of QSOX, which may contribute in crucial ways to protein trafficking and substrate selection, is the segment carboxy-terminal to the Erv domain. As will be described, this region exhibits notable but controlled diversity, and an exploration of this diversity may serve as the basis for insights into QSOX functions and targets.

Herein we provide an updated analysis of QSOX evolution, performed in light of recent structural studies of the QSOX enzyme family. Amino acid sequences were annotated, curated, and subsequently aligned to reveal and compare functional and structural domains and motifs. This study sheds light on the emergence of different QSOX orthologs, paralogs, and isoforms and presents the major evolutionary events that may have led to the contemporary set of QSOX enzymes.

## Results

### QSOX sequences suffer from poor annotation

Many QSOX sequences available in the public databases are currently un- or mis-annotated. Due to the presence of a domain with homology to PDI proteins, the most common mis-annotation is the identification of the QSOX as a PDI. Although both QSOX and PDI act as catalysts of disulfide bond formation, they differ in their cellular localization, domain composition, and electron acceptor. Whereas PDI family proteins are defined by localization to the endoplasmic reticulum and the presence of one or more Trx domains [11], QSOX is defined by the presence of both a Trx domain and an Erv domain [7]. Hence, other annotations such as “Sulfhydryl oxidase like” or “Thioredoxin like,” are similarly insufficient. Another misleading annotation is the numbering of QSOX paralogs. In several instances, the numbering of a QSOX paralog in a given species does not correspond to its counterpart in a second, closely related species. We therefore suggest a coherent and phylogeny-based annotation and numbering for all QSOX sequences identified and used throughout this article. Accession numbers and corresponding annotations are provided as (Additional file 1: Table S1).

### QSOX sequences are widespread, but not universal, in Eukaryota

**Figure 3:**
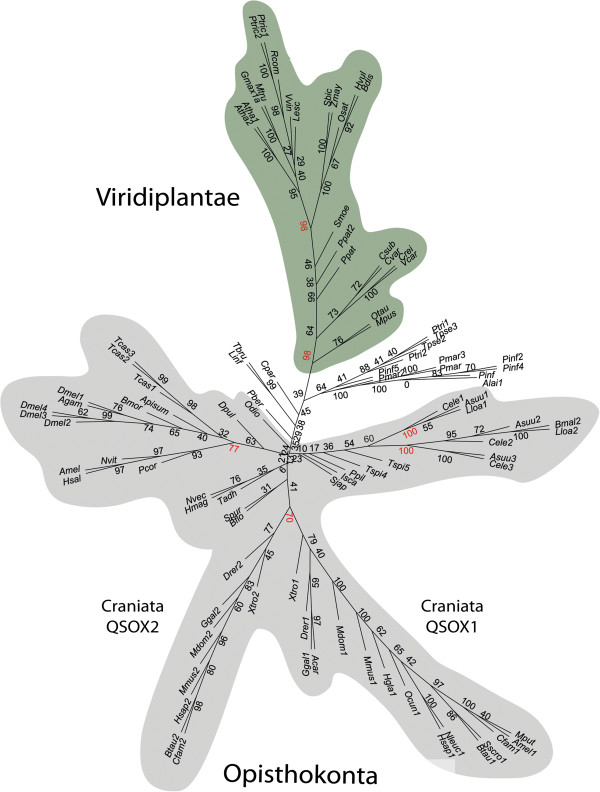
**Consensus maximum-likelihood (ML) tree of QSOX sequences.** Viridiplantae are highlighted in green and Metazoa in gray within the consensus ML phylogenetic tree. The Craniata QSOX1 and QSOX2 branches are indicated. Scores above 70 at major branch-points are highlighted in red.

QSOX sequences were previously identified within a range of eukaryotic organisms, including protists, plants, and animals, but not in fungi [7,12] (Figure 2, Additional file 2: Figure S1). As genomic data continue to accumulate, more QSOX sequences are available for comparative and evolutionary analysis (Figure 3). Of particular interest are organisms that fall at the root of major eukaryotic taxa and enable a better understanding of the primary events leading to the diversity of contemporary QSOX enzymes (Additional file 2: Figure S1).

Stramenopiles, Euglenozoa, and Alveolata are three early branches of unicellular eukaryotes for which QSOX sequences were identified. Within Stramenopiles, QSOX sequences were identified in diatoms. QSOX sequences also appear in Oomycetes, which include several genera of plant pathogens: *Phytophthora*, *Hyaloperonospora*, and *Albugo*. In Euglenozoa, QSOX sequences were identified for *Leishmania* and *Trypanosoma* species, and among Alveolata for *Perkinsus*, *Plasmodium*, and *Cryptosporidium*.

In plants (Viridiplantae) QSOX is found in both green algae (Chlorophyta) and green plants (Streptophyta). Among green algae, QSOX sequences were identified for Chlorophyceae, Mamiellophyceae, and Trebouxiophyceae. Among Streptophyta, QSOX sequences were identified in mosses (Embryophyta) and vascular plants (Tracheophyta). A QSOX sequence was identified for *S. moellendorffii,* member of an ancient lineage at the root of vascular plants in the evolutionary tree. Among the vascular plants, QSOX sequences were found for both monocotyledons and eudicotyledons.

The earliest evidence of a QSOX sequence in Opisthokonta is of the marine choanoflagellate *M. brevicollis*. However, due to a gap in the data affecting the Trx domains, the *M. brevicollis* QSOX sequence could not be retrieved in its entirety, and the sequence was therefore excluded from subsequent analyses. Within Metazoa, a QSOX sequence was identified for the placozoan *T. adhaerens*, which has the smallest animal genome known to date. Among Eumetazoa, QSOX sequences were identified for species of Ctenophora, Cnidaria, and Bilateria (within Acoelomata in Trematoda, within Pseudocoelomata in Nematoda, and within Coelomata). Many QSOX sequences were identified for Arthropoda (both within Chelicerata and Mandibulata) and Chordata, in primitive small invertebrates such as Tunicata and the fish-like cephalochordate *B. floridae*, as well as in Craniata, from fish to humans.

In total, 228 QSOX sequences were identified within the genomes of 132 different species.

### Local QSOX duplications in Viridiplantae contrast with a deep duplication in Craniata

Many organisms contain more than one QSOX gene (Additional file 2: Figure S2). In some species, such duplication events seem to be evolutionarily recent, whereas in others, the duplication occurred deep in the origin of the phylum. Two QSOX paralogs can be found in Craniata from fish to humans (Figure 3), with the exception of several species listed below. The branching at the origin of Craniata means that the human QSOX1 paralog is more similar to fish QSOX1 than to human QSOX2. Within the genomes of *A. carolinensis*, *O. cuniculus*, *M. putorius*, and *S. scrofa*, only the QSOX1 variant was found; in *O. anatinus* only the QSOX2 variant was found. The complementary QSOX paralogs may not have been identified due to poor coverage of the respective genomes.

In addition to the two QSOX paralogs, QSOX sequences have also diversified in Craniata through alternative splicing. In many Craniata, from rodents to humans, an alternative splice variant of QSOX1 has been identified (Additional file 2: Figure S2). This splice variant interrupts the final exon and eliminates the transmembrane segment but preserves all redox-active domains. The splice variation may thus affect processing or localization of the QSOX enzyme. Except for a single occurrence in *M. musculus*, splice variants of the QSOX2 paralog of Craniata have not been identified.

For most arthropods, no paralogs were identified. However, significant exceptions to this generalization are the coleopteran *T. castaneum* and Drosophila, with up to four paralogs in several Drosophila species. The QSOX paralogs of *T. castaneum* do not share the same duplication event as those of Drosophila. In Drosophila, a major duplication event occurred at the root of the genus, giving rise to the QSOX1 paralog present in all Drosophila and to a second branch of QSOX paralogs that underwent several further duplication events with no clear consistency between the species.

Among Nematoda, several duplication events seem to have occurred before the branching of Chromadorea and Enoplea, followed by inconsistent gene duplication and gene loss events.

Among Viridiplantae, QSOX paralogs were identified only within Streptophyta. The duplication events in plants are recent and seem to have occurred locally at the species or genus level. Accumulation of additional QSOX sequences might reveal slightly deeper branching between closely related plants. Nevertheless, as the QSOX paralogs in the two available Arabidopsis species genomes and those of *G. max* do not seem to have emerged from the same duplication event, the branching is not expected to be much deeper.

Among Alveolata, QSOX paralogs were identified for the *P. marinus* species. Due to a lack of genome sequences for closely related species, it is not known how deep this duplication event might be.

Among Stramenopiles, a duplication event may have occurred deep within Oomycetes, followed by multiple inconsistent gene duplication and gene loss events. However, it may be that several relevant paralogs were not identified due to incomplete genome coverage. Another duplication event among Stramenopiles seems to have occurred deep at the level of Bacillariophyta.

This extended catalog of QSOX sequences provides the platform for the following phylogenetic and comparative analyses.

### A single evolutionary path within Viridiplantae contrasts with multiple branching within Metazoa

Several branches seem to emerge from the root of the QSOX phylogenetic tree and evolve apart (Figure 3). Although all neighbor-joining (NJ) and maximum likelihood (ML) trees branch according to the corresponding taxonomic affiliations of the examined species, low consensus scores are obtained at the roots of the major branches. It is only in more recent branches that the distinctions between QSOXs of different species are well supported. According to the consensus ML tree (Figure 3), the Viridiplantae cluster, beginning with the Mamiellophyceae branch, is well distinguished from other QSOX sequences. The distinction between QSOXs of monocotyledons and eudicotyledons is also well supported. Among Metazoa, somewhat reliable scores distinguish QSOXs of Arthropoda and Nematoda after the branching of the *D. pulex* and *T. spiralis* species, respectively. The distinction between QSOX1 and QSOX2 of Craniata is the first to be well supported within Chordata. Lastly, among protists, the Alveolata branch seems to cluster among the Stramenopile branch together with Oomycetes and apart from Bacillariophyta. From all of the above, it is clear that Viridiplantae evolved apart from the various Metazoan branches, although the relative positioning of the branches is poorly supported and hence does not reveal deeper affiliations. Removal of protists from the ML trees slightly improved the distinction between Viridiplantae and several of the Metazoan branches (Additional file 2: Figure S3). Overall, QSOX sequences of Viridiplantae display a continuous path from Chlorophyta to the higher plants in Streptophyta, with a few recent duplication events, whereas highly divergent forms of Metazoa QSOX sequences emerged from a very ancient ancestor.

To assess the evolutionary paths of the QSOX Trx and Erv domains independently, separate ML phylogenetic trees were constructed (Additional file 2: Figures S4 and Figure S5). Similarly to the original ML tree, the ML tree for each domain supports claims for branching order mainly for recent taxonomic affiliations. Nevertheless, important observations can be made upon closer examination. For example, the Trx ML tree shows notable differences from the Erv ML tree along the plant lineage. The Trx ML tree shows the branching of Viridiplantae following Mamiellophyceae, but the clear distinction between monocotyledons and eudicotyledons is missing. Conversely, the distinction between monocotyledons and eudicotyledons is strongly supported by the Erv ML tree, but this tree fails to show the early distinction of plants from other phyla. In other words, the ancient distinction of plant QSOX Trx domains, evident following Mamiellophyceae, was apparently followed by diversification of the Erv domain, particularly notable at the bifurcation of Streptophyta into monocotyledons and eudicotyledons. Therefore, it appears that the Trx and Erv domains of plant QSOXs were subjected to distinct selection events at different points in evolution.

Some differences are also observed between the Trx and Erv domain trees within Metazoa. For example, the Trx ML tree shows support for a distinction between the Craniata QSOX1 and QSOX2 variants well within Mammalia. In the Erv ML tree, however, the diversification of the QSOX2 variant is already well supported at the root of Craniata. QSOX1, in turn, is distinguished in the Erv ML tree from other animal QSOXs at the level of Mammalia. Therefore, it seems that animal QSOX variants underwent a significant evolutionary event that co-affected both their Trx and Erv domains prior to or during the emergence of mammals.

### QSOX redox-active motifs differ between Metazoa and Viridiplantae and show enhanced diversity among paralogs

**Figure 4:**
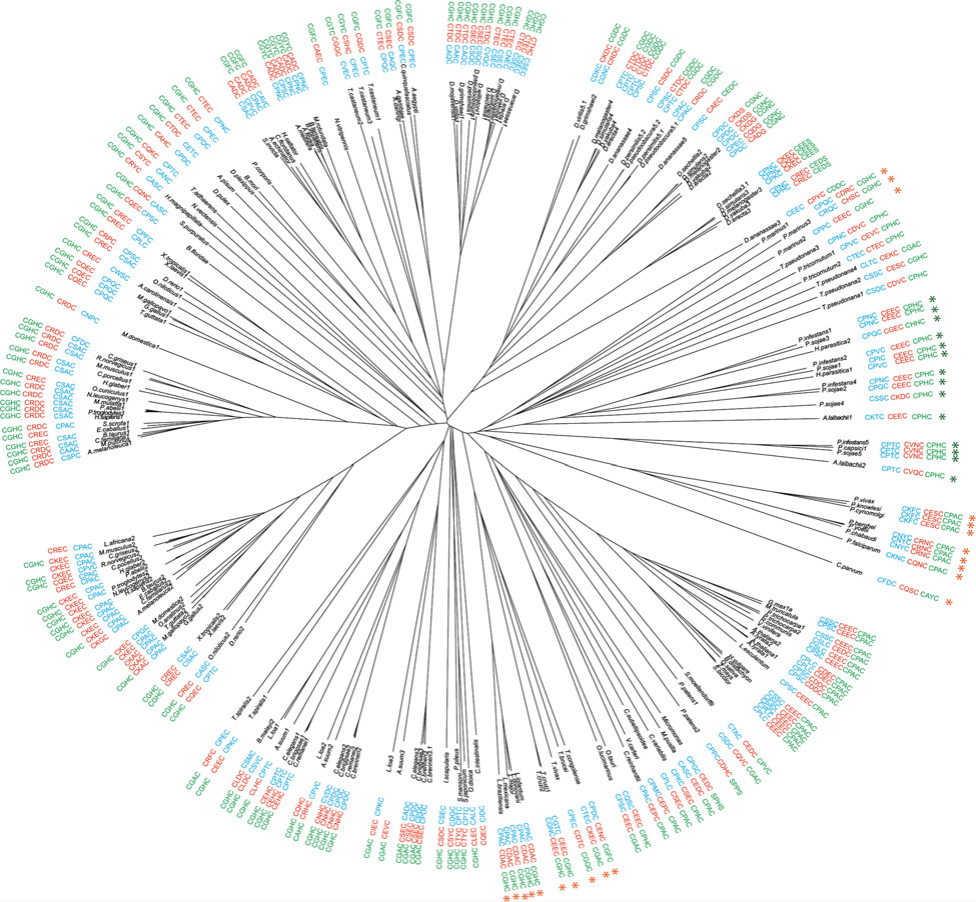
**QSOX CXXC motifs displayed on the neighbor-joining phylogenetic tree.** The Trx-CXXC (green), Erv-CXXC (red), and CT-CXXC (blue) motif patterns are depicted aside the branches of their corresponding QSOX sequences. Green asterisks indicate plant parasites, and orange asterisks indicate animal parasites.

Differences in the redox-active CXXC motifs of the Trx and Erv domains of various organisms were described in a previous review on QSOX [12]. The identities of the intervening residues between the cysteines are expected to play a major role in modulating the redox-potential of the site [13] and may also contribute to interaction specificity. Here we extend the analysis of QSOX CXXC patterns by providing a phylogenetic perspective. Indeed, when we present CXXC motif sequences in the context of the QSOX phylogenetic tree, the motifs can be seen to define distinct groups of QSOX enzymes (Figure 4), and sequence patterns develop along the main branches.

The Trx-CXXC motif shows strong sequence conservation and characterizes major groups of organisms. Specifically, the CGHC sequence is nearly universal in Euglenozoa and Metazoa, with the exception of certain nematodes and arthropods of the Coleoptera, Diptera, and Hymenoptera lineages. Interestingly, among Drosophila, a QSOX paralog with a CGHC pattern coexists with an array of paralogs displaying a CG [DN] C sequence in their Trx-CXXC motifs. Similarly, in nematodes, QSOXs with a CGHC patterns are found together with a paralog exhibiting a CGAC pattern. To the extent possible, we assigned the designation QSOX1 to paralogs containing the CGHC pattern dominant in Metazoa QSOXs. Among Viridiplantae and the Apicomplexa branch of alveolates, the CPAC pattern is the most abundant, whereas Stramenopiles typically have a CPHC pattern. It is interesting to note that a CPXC pattern is rare among PDI family Trx domains, for which the CGHC pattern is prominent, even in PDIs from plants [14]. Hence, the distinction between the three major patterns (CGHC, CPHC, and CPAC) for Trx redox-active motifs in QSOX enzymes is independent of trends for CXXC motifs in PDI family proteins.

The Erv-CXXC patterns are more diverse than the Trx-CXXC patterns. For example, five organisms at the base of Metazoa, *T. adhaerens*, *N. vectensis*, *H. magnipapillata*, *S. purpuratus*, and *B. floridae*, all share the common Trx-CXXC sequence CGHC but have the Erv-CXXC sequences CQKC, CSYC, CRYC, CQNC, and CQEC, respectively. In Craniata, variation in the Erv-CXXC motif follows the taxonomic lineage. Specifically, both QSOX1 and QSOX2 of lower taxa often contain a CREC pattern, whereas the patterns diverge in higher taxa such that CRDC becomes dominant for QSOX1 and CKEC for QSOX2. In general, Erv-CXXC patterns delimit narrower groups of organisms than do Trx-CXXC patterns and vary in most instances of paralogs. Even in some recent duplications in which paralogs retain high sequence identity, the Erv-CXXC motifs differ. For example, the Erv-CXXC motifs have diverged following the independent duplications in Arabidopsis (CEEC *vs.* CEDC) and Trichocarpa (CDDC *vs.* CDEC).

Consideration of the various QSOX CXXC patterns in light of the phylogenetic tree allowed us to distinguish between diversity that implies the absence of constraints from diversity that suggests adaptation. In particular, the strong conservation of Trx-CXXC patterns within different lineages suggests tight constraints along these branches, despite the prominent differences in patterns between lineages. A phylogenetic perspective also aids in discrimination between primary and auxiliary paralogs. The primary paralog is present in closely related species and tends to exhibit the same CXXC patterns, whereas auxiliary paralogs greatly increase the sequence diversity of the motif. Finally, a phylogenetic approach allows speculation regarding the primordial patterns that diversified into those exhibited by contemporary organisms. For example, the occurrence of CPAC in the Trx-CXXC of plants may have arisen from a primordial CPHC pattern, still exhibited by Stramenopiles and even in two occurrences of Chlorophyta.

### Intron positions do not reveal a common imprint between Viridiplantae and Metazoa

**Figure 5:**
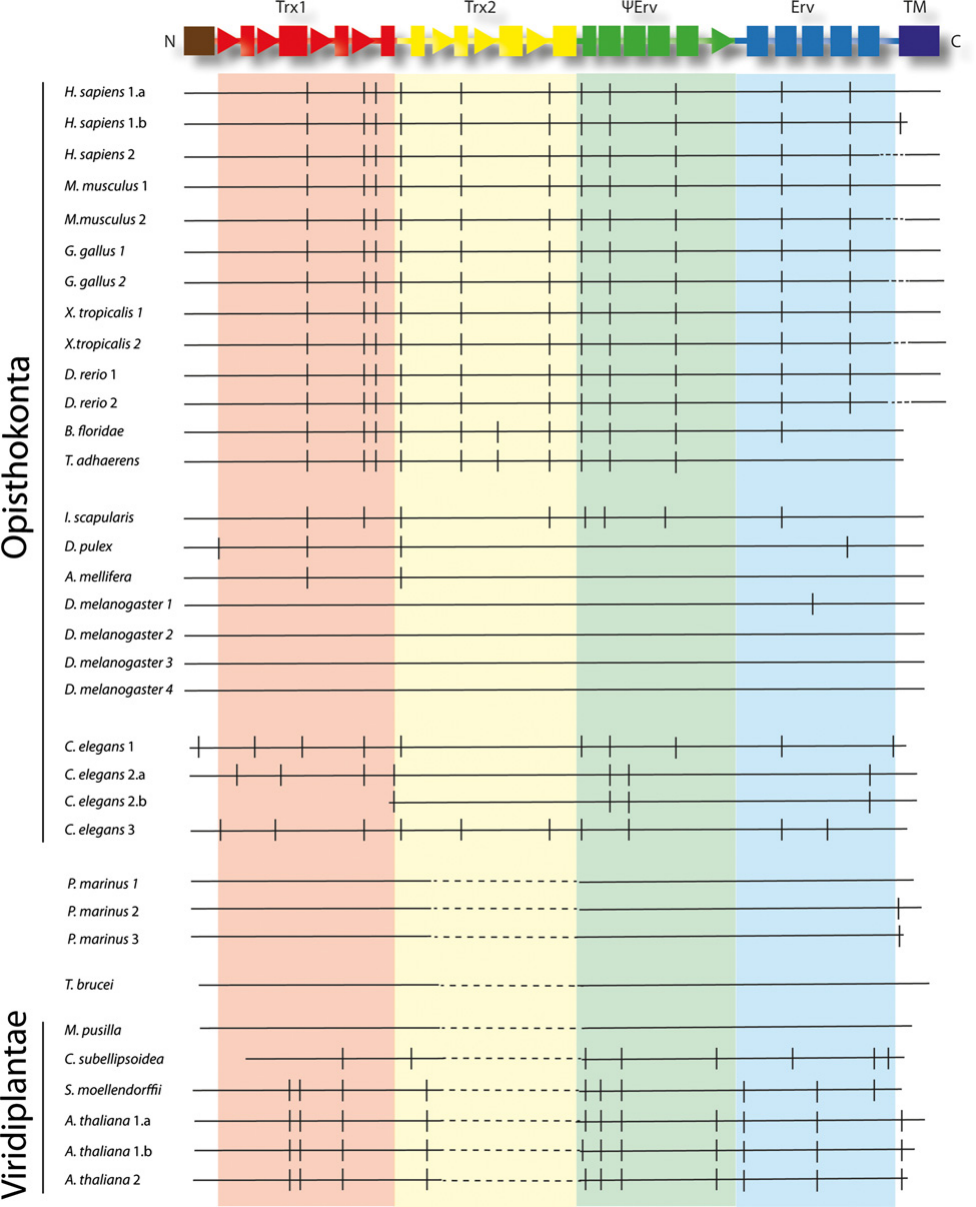
**Intron positions of QSOX sequences along the secondary structure.** Predicted intron positions in representative QSOX genes are displayed. The QSOX domain organization and secondary structure scheme appears at the top of the panel. Dashed lines represent regions absent in the corresponding QSOX sequence.

The differing numbers of Trx-fold domains in Metazoa *vs.* other QSOXs, the absence of QSOX from fungi, the different motif sequences, and the ambiguities at the roots of major branches in QSOX phylogenetic trees prompted us to consider whether contemporary QSOX enzymes may be descended from more than a single fusion event linking a Trx-fold to an Erv domain. To search for evidence to the contrary, we inspected QSOX intron positions through various taxa (Figure 5). It has been previously observed that *H. sapiens* share intron positions with *A. thaliana* in certain orthologous genes. Even if the ancestral sequence endured several intron gains and losses, a primitive imprint could still be distinguished [15]. Inspection of QSOX intron positions revealed diversification between phyla, with apparently no common intron positions among Viridiplantae and Metazoa. Some closely situated or ambiguous positions will be discussed for clarification. In one case, an intron marks the end of the α2 helix of the Trx domain among many Metazoa, whereas an intron occurs at the beginning of the helix close to the Trx-CXXC active site in Viridiplantae. The following intron in Viridiplantae is at the beginning of the α3 helix, whereas in Metazoa the intron is at the end of the β4 strand. In the ψErv domain, an intron is located before the α1 helix in both Viridiplantae and Metazoa, but the lack of the Trx2 domain as well as any sequence conservation makes it difficult to conclude from this one coincidence that plant and animal QSOX have a common origin. Further on, two introns are located before and after the α2 helix in Viridiplantae, whereas in Metazoa the intron is located within the α2 helix. In summary, no residual signature of a common evolutionary origin between plant and animal QSOX was detected in the pattern of intron positions. This result does not imply that QSOX enzymes from different kingdoms lack a common ancestor, only that evidence for a common ancestor is lacking in the intron patterns.

### The ψErv/Erv module, strongly characteristic of QSOX, contrasts with a Trx module only weakly differentiated from PDI family domains

We then sought more insight into the potential origins of the Trx and Erv domains to assess the likelihood of various scenarios that may have produced the current QSOX family. A comparison of the QSOX Trx and Erv domains with other proteins containing only one or the other of these domains demonstrated that the QSOX ψErv/Erv module is clearly distinct from all other known Erv enzymes. The most widespread member of the Erv family is Erv1, a mitochondrial enzyme found broadly throughout eukaryotes, including yeast. Erv1 is a symmetric dimer, in which the two copies of the Erv domain interact. This arrangement differs significantly from QSOX, in which the Erv domain forms a pseudodimer with the ψErv domain and is therefore inaccessible for true dimerization. The presence of the ψErv region immediately upstream of the Erv domain in QSOX, as well as the existence of a third, carboxy-terminal CXXC motif (CT-CXXC), are unique characteristics of the QSOX subfamily of Erv enzymes that distinguish them definitively from other Erv domains. Remarkably, the CT-CXXC motif, despite being dispensable for enzyme assays *in vitro*[8], is as highly retained as the two catalytic CXXC motifs of QSOX. In summary, the available data suggest that the ψErv/Erv modules in all contemporary QSOX enzymes share a common ancestor.

In contrast to the clear distinction between the ψErv/Erv module of QSOX enzymes and Erv domains of other proteins, the distinction between Trx domains of QSOXs and PDI family proteins is less evident. The strong structural similarity among Trx domains from PDI proteins, and between PDI and QSOX, makes the identification of defining features of Trx domains in one protein family *vs.* the other quite challenging. Unlike the extension containing the CT-CXXC motif of QSOX, which is a distinguishing characteristic of its Erv domain, the CX_6-8_C disulfide in the QSOX Trx1 domain is found also in PDI proteins. No secondary structure element, loop, or other structural feature appears to differentiate QSOX Trx domains from PDI Trx domains. Furthermore, the large and ancient PDI family provides a rich source for both Trx and juxtaposed Trx1/Trx2 domains from which the Trx modules in plant and animal QSOXs may have been derived. Focusing on amino acid sequences rather than structural features, the CGHC and CPAC patterns, found in the Trx domains of major QSOX lineages, are observed in various PDI proteins as well. Interestingly, the CGHC pattern is strongly dominant among PDI proteins that contain non-catalytic Trx domains downstream of a catalytic one, just as the CGHC pattern is characteristic of QSOXs that contain a catalytic/non-catalytic arrangement of Trx domains. In turn, CPXC patterns are frequently observed in PDI family proteins that lack non-catalytic Trx domains, analogous to plant and protist QSOXs.

Despite the similarities between QSOX and PDI Trx modules, in all phylogenetic trees constructed to include both sets, QSOX Trx domains clustered separately, although with very low scores at the root (Additional file 2: Figure S6). Further inspection of QSOX sequences compared to PDIs revealed an amino acid position that differs consistently between the two protein families. Two residues upstream of the CXXC motif in the Trx domain, a proline that is found nearly universally in PDI proteins is replaced by, most often, a serine or threonine in metazoan QSOXs or a histidine in plant QSOXs (Additional file 2: Figure S7). A proline is found only in select QSOX enzymes, such as those from *O. tauri*, *M. pusilla*, and *P. infestans*. The phylogenetic trees and the pinpointing of particular residues suggests that subtle sequence differences do distinguish QSOX and PDI Trx domains, consistent with either divergent evolution of QSOX Trx domains from a single precursor or convergence of distinct QSOX Trx domain sequences due to shared pressure to function in conjunction with the ψErv/Erv module.

In either scenario, the question remains whether the fusion of Trx and Erv domains has a common purpose across all organisms that display this fusion. In particular, is the fusion indicative of a shared biochemical mechanism? The availability of QSOX crystal structures [9] allowed for an analysis of QSOX sequences against the backdrop of the three-dimensional structures. The ensuing analysis of conservation at the level of QSOX domains was conducted to shed more light on the evolution of fundamental structural and mechanistic elements of the QSOX enzyme.

### Conservation at the Trx-Erv domain interface suggests a conserved electron transfer mechanism

**Figure 6:**
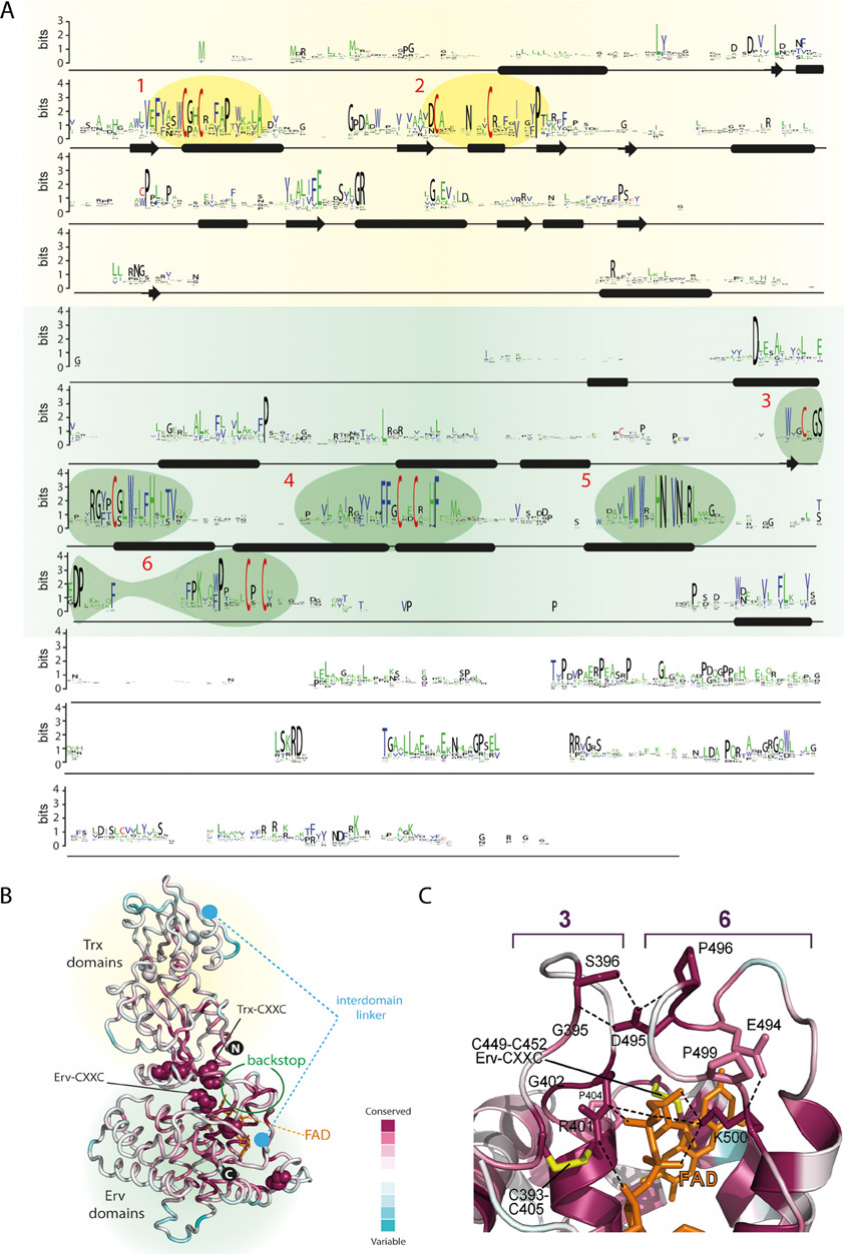
**Conservation profiles of QSOX sequences.** The oxidoreductase module is on a pale yellow background, and the sulfhydryl oxidase module is on a green background. (**A**) QSOX sequence logo. Highly conserved regions discussed in the text are highlighted and numbered. (**B**) Conservation profile displayed on the QSOX structure. The hybrid human QSOX1 structure was generated as described in the Methods. Conserved residues are in magenta, and variable residues are in cyan, as depicted in the color scheme at the bottom right. Cysteine residues are indicated in space-filling representation, and the Trx- and Erv-CXXC motifs are labeled. The interdomain linker, missing from the structure, is depicted with dashed lines between its endpoints (cyan disks) in the two modules. The backstop is marked by a green circle. (**C**) Structural details of two loops in conserved regions #3 and #6. Together these loops constitute the conserved backstop in the ψErv/Erv domains against which the Trx1 domain docks. The color scheme used to indicate the level of conservation is the same as in panel **B**. The FAD is depicted in orange and the disulfides in yellow. Potential hydrogen bonds or salt bridges are indicated by dashed lines. Amino acid residue numbering is according to the *H. sapiens* QSOX1 sequence.

As described above (Figure 1), QSOX enzymes are characterized by the fusion of a Trx domain to an Erv domain bridged by non-catalytic structural elements. The two catalytic domains show the strongest sequence similarity. Excluding core secondary structure elements such as the central Trx1 β-strands, six regions of particularly notable similarity are detected in QSOX, as highlighted in a sequence logo built from all available QSOXs (Figure 6A). Comparison of these regions with the QSOX structure allows the function of each to be assessed and emphasizes the importance of the interaction between domains.

The first two segments of conservation correspond to the Trx1 redox-active site and the CX_6-8_C disulfide-bonded loop adjacent to it in the tertiary structure. Together, these two regions constitute the interface between the Trx1 and the Erv domains when they are packed against one another in the electron-transfer intermediate [9]. To better appreciate the conservation at the contact site, conservation scores were mapped onto the QSOX structure (Figure 6B). Apart from the Trx-CXXC motif, the most conserved residue in these two segments is a proline at the beginning of the β4 strand. This proline, present in many Trx-fold proteins and found structurally in the *cis* configuration, was previously suggested to contribute to substrate binding [16] or shown to facilitate substrate release [17] or inhibit metal binding by the reactive thiolate-based active site of thioredoxins [18]. Following the *cis*-proline, there is poor conservation at the sequence level in QSOX, though at the structural level the domain is predicted to retain an α4 helix.

The third region of conservation (Figure 6C) is another disulfide bonded loop at the amino-terminal junction of the Erv domain (Cys449-Cys452 in the *H. sapiens* QSOX1 sequence). Within the eleven residues between the cysteines in this loop is a basic residue (Arg401 in the *H. sapiens* QSOX1 sequence) that makes an electrostatic interaction to the phosphates in the FAD cofactor. The remainder of the loop projects out from the Erv helical bundle to contribute, together with the sixth region of conservation detailed below, to a structural element that has been described as a “backstop,” since it seems to constrain the Trx1 domain sterically as it docks against the Erv domain during interdomain electron transfer. The presence of this backstop is one of the major differences between QSOX Erv domains and other Erv enzymes. This difference may reflect the structural contexts of the di-cysteine motifs that interact with the Erv-CXXC in QSOX compared to other Erv enzymes. Specifically, the QSOX Trx domain, from the opposite end of the multi-domain protein, interacts with the QSOX Erv domain [9], whereas di-cysteine motifs on flexible regions of polypeptide tethered locally interact with the FAD-proximal disulfide of stand-alone Erv domains [19,20]. The sequence conservation in QSOX extends from the backstop loop through the first three turns of the α1 helix in the Erv domain, where a tryptophan and histidine project from the same side of the α1 helix to form part of the FAD binding site.

The fourth conserved region in QSOX comprises the Erv redox-active di-cysteine motif. In addition, a highly conserved histidine and phenylalanine follow the CXXC motif in the α3 helix of the Erv domain. The imidazole side chain of the histidine is surface-exposed and interacts with the polar edge of the FAD isoalloxazine. The phenylalanine, together with the α1 helix tryptophan mentioned above, contributes to the hydrophobic pocket occupied by the non-polar edge of the isoalloxazine.

The fifth conserved region is the α4 helix in the Erv-domain helical bundle, which contains a set of side chains that contacts the adenine portion of the FAD. Specifically, a histidine and two asparagines are hallmarks of Erv domains [21]. The α4 histidine interacts with the conserved histidine from the α1 helix, and the two imidazole rings, in a planar arrangement, stack against the FAD adenine ring.

Finally, the sixth conserved segment (Figure 6C) comprises the CT-CXXC motif. The CT-CXXC disulfide is immediately downstream of another basic residue (Lys500 in the *H. sapiens* QSOX1 sequence) in the vicinity of the FAD phosphates and an aromatic residue that packs against the outer face of the FAD adenine. Non-QSOX Erv domains have these same two functional residues, but in a simpler structural context: they are present in a KXXF/Y motif on the approximately ten-residue linker that directly connects the fourth and fifth helix in the Erv bundle. In QSOX, there are insertions both upstream and downstream of the basic/aromatic residue pair. Upstream is a stretch of about ten residues that constitute the second half of the backstop by packing against the third conserved segment (Figure 6C). Downstream of the basic/aromatic pair is the CT-CXXC and a weakly conserved loop preceding the fifth helix in the domain. The presence of the loop between the CT-CXXC and the fifth helix is conserved, though its composition less so. The function of this loop in the QSOX structure or mechanism is still unclear.

In summary, regions of highly conserved sequences in QSOX correspond to 1) important redox-active or structural disulfide bonds, 2) residues in direct contact with the FAD cofactor, and 3) features that appear to contribute to the interaction of the Trx and Erv domains but do not appear to be essential for the folding or fundamental catalytic activity of each module in isolation. In particular, the backstop in the ψErv/Erv module, contributed by regions #3 and #6 described above, is a prominent feature that appears to promote domain docking for electron transfer.

### Evidence of functional constraints within regions of high sequence variability

Inspection of sequences that are poorly conserved may also shed light on aspects of QSOX function. An apparently poorly conserved region that nevertheless is likely to be crucial for QSOX activity is the linker between the Trx domain(s) and the ψErv region. The sequences of this linker are highly variable, but the lengths fall into a narrow range: about 20–35 amino acids between the predicted ends of the flanking secondary structure elements. A rough conservation of linker length is consistent with the proposed role of the linker in providing flexibility for relative domain rotations in QSOX function, whilst tethering the two interacting domains with high effective concentration [9]. Although some outliers appear to contain linkers of up to a hundred amino acids, these may arise from erroneous predictions of splice sites based on genome sequences.

**Figure 7:**
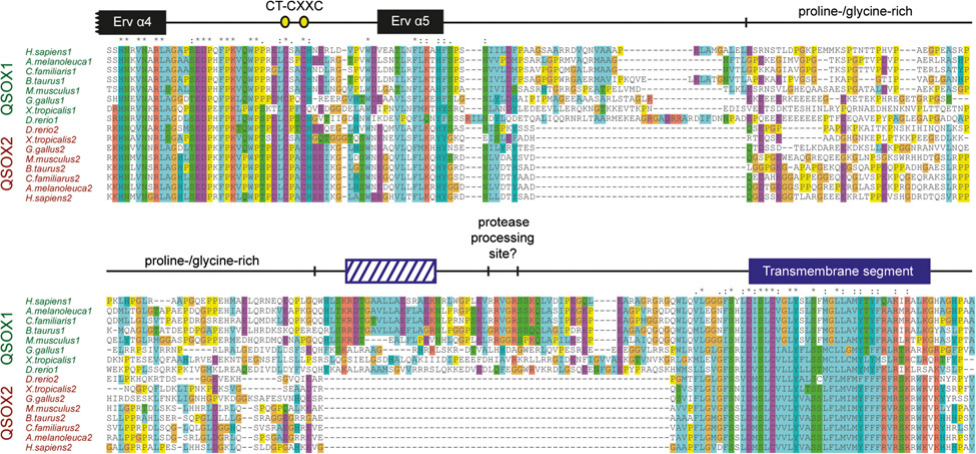
**Amino acid sequence alignment of the C-termini of Craniata QSOX1 and QSOX2.** Representative QSOX1 and QSOX2 sequences are shown. The positions of helices (α4, α5) from the Erv domain are indicated as black rectangles. The blue diagonal-hashed rectangle indicates a segment predicted to be helical. A stretch of basic amino acids downstream of the predicted helix is speculated to be a protease cleavage site for QSOX1 processing based on mass spectrometry fingerprinting of secreted QSOX1 (Ilani T, Alon A, Grossman I, Horowitz B, Kartvelishvily E, Cohen SR, Fass D: A secreted disulfide catalyst controls extracellular matrix composition and function, submitted) and the preponderance of basic amino acids in recognition sites for secretory pathway proteases such proprotein convertases.

Other positions in QSOX, in particular the N- and C-terminal extremities, appear variable in overall sequence alignments of the enzyme family. Indeed, there appear to be few constraints on the sequence upstream of the QSOX Trx domain. The apparent variability near the C-terminus, however, masks a certain underlying homogeneity. In particular, strong sequence conservation within taxonomic groups is observed in the region between the Erv domain and the transmembrane segment, and though QSOX paralogs differ from one another here, the differences are often retained across species. For example, Craniata QSOX1 and QSOX2 show a characteristic difference in the number of amino acids linking the Erv domain to the membrane (Figure 7). An extra segment present in QSOX1 contains a predicted helix followed by a set of basic residues that may be a protease processing site (Ilani T, Alon A, Grossman I, Horowitz B, Kartvelishvily E, Cohen SR, Fass D: A secreted disulfide catalyst controls extracellular matrix composition and function, submitted). The conserved differences between QSOX1 and QSOX2 suggest that the membrane-proximal region may contribute to differing function, localization, or processing of the QSOX1 and QSOX2 paralogs.

## Discussion

The structures and dynamic relationship of the component parts of the QSOX enzyme, as well as the steps in its internal electron relay, have been recently determined [9]. Furthermore, the targeting and localization of QSOX to the Golgi apparatus and extracellular environment, at least in mammals, is now evident [4,6] and (Ilani T, Alon A, Grossman I, Horowitz B, Kartvelishvily E, Cohen SR, Fass D: A secreted disulfide catalyst controls extracellular matrix composition and function, submitted). With its unique domain composition, localization, and functional independence, it is likely that QSOX acts in a fundamentally different pathway or pathways than other disulfide forming enzymes in the cell. Although the complement of disulfide catalysts differs between organisms, the presence of one or more disulfide forming enzymes in the ER is considered necessary for the central role of this organelle in oxidative protein folding. QSOX, outside the ER, appears not to play a general role in folding the pool of secretory proteins, but may rather be dedicated to particular targets.

Trx-fold domains are universal in prokaryotes, archaea, and eukaryotes, but the fusion of a Trx domain with an Erv sulfhydryl oxidase domain is unique to the eukaryotic cell. QSOX is clearly not essential for eukaryotes, however, as yeast appear to lack the enzyme entirely. One may speculate that QSOX activity is related to multicellularity, which is in keeping with an observed role in extracellular matrix formation in animals (Ilani T, Alon A, Grossman I, Horowitz B, Kartvelishvily E, Cohen SR, Fass D: A secreted disulfide catalyst controls extracellular matrix composition and function, submitted). According to this model, the presence of QSOX in unicellular parasites such as trypanosomes and the resemblance of the Trx domain redox-active site of parasite QSOXs to that of their hosts (Figure 4) suggest that host proteins may be the targets of the pathogen-derived disulfide catalyst. Complicating the emphasis on multicellularity, however, is the observation that QSOX is found in unicellular green algae and in diatoms. The possibility therefore remains open that QSOX evolved to oxidize different target proteins on different pathways and for different purposes in plants, animals, and other species, rather than playing a universal biological role in all the species that encode it.

The most evident structural difference between plant and animal QSOXs is their domain composition. A number of different models can be discussed for how the QSOX family acquired its inhomogeneous domain content. If we assume that all current QSOX enzymes derived from the same original fusion between Trx-fold and ψErv/Erv elements, then either the fusion involved a single Trx domain, and metazoans subsequently gained an additional Trx domain, or the fusion occurred with a tandem Trx1/Trx2, and non-metazoans subsequently lost the Trx2 domain. From our analysis, however, we cannot rule out a model in which two independent fusion events of the ψErv/Erv unit with either a single Trx domain or tandem Trx domains led to the set of contemporary QSOXs. Another model that can be considered is one in which a universal QSOX precursor arose by fusion of a single Trx domain with the ψErv/Erv module, and the Trx domain was then replaced by a tandem Trx1/Trx2 early in the evolution of metazoans. In the latter two models, a subsequent modest convergence of QSOX Trx sequences, reflecting shared pressure to function in an electron relay with the ψErv/Erv module, would then explain the weak distinction of both plant and animal QSOX Trx domains as a group from PDI Trx domains. It should be noted that each of the models presented involves a minimum of two major fusion/deletion/substitution events in evolution. However, an independent fusion of the ψErv/Erv module with either a single Trx *vs.* tandem Trx domains on different lineages may nevertheless be less parsimonious, since it requires a ψErv/Erv module to be retained and available for both fusions at distinct points in time. In all examined sequences, the ψErv/Erv unit of QSOX enzymes is clearly distinct from single-domain Erv enzymes present in these same organisms, and there is little doubt that this module of QSOX evolved divergently from a single precursor. In particular, the backstop region and the CT-CXXC motif are present universally in QSOX enzymes but lacking in other Erv proteins. In the absence of evidence that the ψErv/Erv unit has a function on its own, it may be unlikely that it existed in primordial genomes until it was independently incorporated into two evolutionarily distinct fusion proteins with different Trx modules.

**Figure 8:**
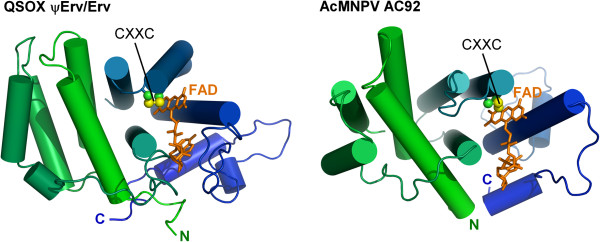
**The QSOX ψErv/Erv module resembles a baculovirus Erv enzyme.** The structure of the *H. sapiens* QSOX sulfhydryl oxidase module [11] is compared with one subunit of the baculovirus Ac92 sulfhydryl oxidase dimer [22]. Helices are represented as cylinders, the FAD is in orange sticks, and the FAD-proximal redox-active disulfide is indicated in ball-and-stick format and labeled CXXC.

In considering the origin of the the ψErv/Erv arrangement, it should be noted that a similar juxtaposition of non-catalytic and catalytic Erv-like folds has been observed outside the QSOX subfamily. The sulfhydryl oxidase from the baculovirus *Autographa californica* multiple nucleopolyhedrovirus (AcMNPV) [22] (Figure 8) is also the product of an apparent gene duplication and fusion, but there is no evidence that QSOX and the AcMNPV sulfhydryl oxidase arose from the same event. The viral enzyme lacks the CT-CXXC motif, and, outside of its gross topology, bears little detailed resemblance to QSOX. Furthermore, the baculovirus enzyme does not contain a Trx-fold domain. Nevertheless, the importance of nucleocytoplasmic large double-stranded DNA viruses (NCLDVs) in the evolution of early eukaryotes and the presence of an Erv sulfhydryl oxidase as a core gene in NCLDVs leaves open the possibility of a viral contribution to the origin of QSOX.

In contrast to the ambiguities regarding the sources and evolution of the QSOX domains, the comparative analysis performed against the backdrop of the QSOX enzyme structure suggests that the QSOX biochemical mechanism is well-understood and conserved throughout the evolutionary tree. In particular, the transfer of electrons from the Trx to the Erv domain (Figure 1) by a dithiol/disulfide exchange step appears to be characteristic of QSOX enzymes. As noted above, the required interaction between the Trx and Erv domains is strongly supported by conservation of the “backstop” region, a feature absent from Erv family enzymes that do not receive electrons at their FAD-proximal disulfide directly from a Trx-fold domain. In addition, the conservation of a region with variable sequence but relatively uniform length between the oxidoreductase and sulfhydryl oxidase modules supports the notion that the orientation between the Trx and Erv domains is flexible but the effective concentration of the two redox-active domains is constrained.

Given the support from sequence analysis for a universal interdomain electron transfer mechanism in QSOX, it is therefore puzzling that a biochemical study of recombinant *A. thaliana* QSOX failed to detect this step [23]. The recombinant enzyme appeared to be well-folded, the Trx redox-active motif could readily accept electrons, and the Erv domains displayed robust sulfhydryl oxidase activity. Nevertheless, the Trx domain did not appear to transfer electrons to the Erv domain in the plant enzyme. The lack of interdomain electron transfer in recombinant plant QSOX cannot be explained merely by the absence of the Trx2 domain, as recombinant *T. brucei* QSOX, also lacking a Trx2 domain, showed effective interdomain dithiol/disulfide exchange and activity virtually indistinguishable from mammalian QSOX homologs [8,9,24]. This conflict between the *in vitro* enzymology of plant QSOX and the structure-guided sequence analysis presented here suggests that much remains to be discovered regarding the *in vivo* context of plant QSOX and the manner by which its localization, post-translational modification, and processing may affect its ability to carry out its anticipated enzymatic cycle. The puzzling biochemistry of plant QSOXs may be related to the observation that the Trx and Erv domain sequences appear to have diverged in independent steps along the plant lineage, suggesting that the domains were subjected in plants to other pressures besides their mutual interaction.

The open questions regarding plant QSOX enzymology emphasize that, sterically and electrostatically, the interdomain redox relay that occurs in QSOX is not trivial. It has been noted that redox relays tend not to involve direct transfer of electrons between Trx domains [25], a generalization that may apply, in most cases, to transfer between Trx and Erv domains as well. The reason for this apparent prohibition is the unfavorable apposition of helix dipoles as the Trx- and Erv-CXXC motifs, both at the amino termini of helices, interact. The elucidated mechanism of QSOX enzymes [8,9] nevertheless includes direct electron transfer between helix amino-termini, indicating that such an event, while rare, does occur in a natural biological pathway. This unusual interaction is likely to be compensated by other features of the protein-protein interaction interface or more globally within the enzyme. In particular, the tethering of the Trx and Erv modules to one another to increase their effective concentration and the presence of the “backstop” may be necessary, but not sufficient, to promote the interaction. Further studies of plant QSOX, including high-resolution structural information, will provide a wider view of QSOX evolution and correct any biases acquired by a focus on metazoan and parasite representatives of the family.

## Conclusions

Despite certain marked differences among QSOX sequences from different phyla, strong similarities within the redox-active domains suggest that fundamental aspects of the mechanism described for metazoan QSOX enzymes are shared among QSOXs throughout Eukarya. Based on sequence alignments of QSOX enzymes from diverse species and an understanding of the physical basis for interdomain electron transfer derived from QSOX crystal structures [9], not only the presence of the Trx and Erv domains but also aspects of their interaction interfaces appear to be shared in common. Regardless of the possibly differing substrate sets available to QSOX enzymes in different species, and the potentially different pathways in which these substrates function, there is validity to the annotation of all detected Trx-Erv domain fusions as QSOX enzymes. QSOX enzymes appear to be characterized not only by a Trx domain and an Erv domain at opposite ends of a multi-domain protein, but also by the potential to carry out a complete electron transfer pathway from substrate dithiols to the terminal electron acceptor, molecular oxygen.

## Methods

### Identification and curation

The NCBI BLAST tool (http://www.ncbi.nlm.nih.gov/BLAST/) was used with various full length QSOX protein sequences to exhaust the available databases at NCBI, at both the nucleotide (nt, refseq_rna, refseq_genomic, and est) and protein (nr, refseq_protein, swissprot, and pdb) levels. When identified, expressed sequence tags (ESTs) were assembled into contigs and compared to the corresponding genomic DNA. Identification of exon boundaries and consensus splice sites as well as of sites of transcription termination was done by comparison of genomic DNA and cDNA sequences. All retrieved sequences were manually curated. Splicing variants of QSOXs were identified by visual inspection.

It should be noted that the resemblance of the QSOX Trx and Erv domains to other thioredoxins and sulfhydryl oxidases made the identification more complex, especially for short (*i.e.,* lacking one of the domains) and isolated (*i.e.,* lacking phylogenetically close homologs) sequences. Sequences were therefore accepted as QSOX if they had both a Trx1 and an Erv domain, as the existence of both domains is a defining characteristic of QSOX sequences. In several instances, segments of the Trx or Erv domains were missing, and the sequence was confirmed as QSOX by comparison with a closely related homolog. In all these instances, strong homology could be observed along the sequences and not only at *loci* of the remaining domain. There were no predetermined cutoffs, but the minimal similarity was 68%, calculated for L. loa QSOX3 (lacking parts of its Trx and Erv domains) and A. suum QSOX3.

### Alignment and construction of phylogenetic trees

Protein sequences were aligned using the ClustalX program with default settings [26]. The neighbor-joining phylogenetic trees, used to display paralogs and CXXC patterns, were constructed using the embedded algorithms of the ClustalX program.

To assess evolutionary events from QSOX sequences, more robust maximum likelihood (ML) trees were constructed. For the ML trees of the QSOX Trx and Erv domains, the following patterns were generated: Trx: X(5)-[DGEILQKVNRM]-x(5)-[WFYKSGT]-[CS]-x(2)-[CS]-x(4)-[PRSDETGAKHN]-x(10,35)-C-x(5,9)-C-x(8)-P-[AGTSLMFRHKNQ]-X(9), Erv: X(30)-[TNPSLF]-C-[GSTA]-X(18,54)-C-x(2)-[CSG]-x(2)-[HNKEY]-[FIL]-x(40,116)-[WFY]-x(5)-C-x(2)-C. Truncated QSOX sequences (indicated in Additional file 1: Table S1) were omitted from the dataset used for the analysis. To avoid over-representation on particular branches, one representative species from each genus was included (indicated in Additional file 1: Table S1). Concatenation of the Trx and Erv domains was used to build the QSOX ML tree. All ML trees were constructed using the Phylip package [27]. First, 100 bootstrap replicates were generated using the seqboot program (seed set to 9). The ML trees were then generated using the proML program with the Dayhoff and Jones-Taylor-Thornton (JTT) probability models (seed set to 9 and jumble to 3). Lastly, consensus trees were established using ‘Consense,’ available in the Phylip package. Figures were created using FigTree.

### Highlighting conserved residues on QSOX

A number of QSOX crystal structures are available [9,10]. These include the *H. sapiens* structure determined in two fragments (PDB codes 3Q6O and 3LLK), a mutant *M. musculus* four-domain structure (PDB codes 3T58 and 3T59), and wild-type and mutant *T. brucei* structures (PDB codes 3QCP and 3QD9). A structure model for a “closed” or “docked” state of *H. sapiens* QSOX1 was constructed by superposition of the structures of the oxidoreductase [9] and sulfhydryl oxidase [10] portions of *H. sapiens* QSOX, determined separately by X-ray crystallography (PDB codes 3Q6O and 3LLK respectively), onto the structure of a mutant of *M. musculus* QSOX designed to mimic the intermediate in interdomain electron transfer (PDB code 3T58) [9].

To improve the accuracy of the QSOX sequence logo, secondary structures were predicted using PSIPRED [28] and used to manually adjust the alignment in certain cases. In some sequences that lack the Trx2 domain, segments of the ψErv domain were erroneously pulled into the gap, but these segments could be returned readily to their proper positions based on the high helix content of the ψErv domain and its conservation on the structural level. Such adjustments were unnecessary in other analyses, which were based on alignments that excluded the Trx2 domain. Logos were created using WebLogo [29]. The QSOX multiple sequence alignment was then aligned to the atomic coordinate file using the ConSurf server [30], resulting in a color coded scheme of conserved residues mapped onto the model of the *H. sapiens* QSOX1 closed state structure.

## Competing interests

The authors declare no competing interests.

## Authors’ contributions

KLW and DF conceived the study. KLW searched the databases for QSOX sequences. SBD curated the sequences. KLW planned and conducted the analysis of the data. SBD contributed expertise in phylogenetics. KLW and DF analyzed the findings and wrote the paper. All authors read and approved the final manuscript.

## Supplementary Material

Additional file 1: Table S1 Accession numbers and annotations of QSOX sequences identified in the NCBI databases. Truncated sequences and sequences included in the phylogenetic ML study are indicated. Click here for file

Additional file 2: Figure S1 Taxonomic classification of species for which a QSOX sequence was identified. The given classification is based on the NCBI taxonomy browser. **Figure S2.** QSOX orthologs, paralogs, and splice variants in various taxonomic classifications. To emphasize the hierarchy between orthologs and paralogs, Neighbor-joining (NJ) trees were constructed for QSOX sequences of Alveolata, Nematoda, Stramenopiles, Viridiplantae, Arthropoda, and Chordata. **Figure S3.** Consensus maximum likelihood tree of QSOX sequences, protists excluded. In an attempt to improve scores at the root of major branches, QSOX sequences of protists were removed from the dataset. **Figure S4.** Consensus maximum likelihood tree of the Trx domain of QSOX sequences. The Trx domains of QSOX sequences were retrieved using the pattern detailed in the methods section. Abbreviations correspond to species as listed in Figure S3. **Figure S5.** Consensus maximum likelihood tree of the Erv domain of QSOX sequences. The Erv domains of QSOX sequences were retrieved using the pattern detailed in the methods section. Abbreviations correspond to species as listed in Figure S3. **Figure S6.** Example of a neighbor-joining tree of the Trx domain/s of QSOX and PDI sequences. PDI sequences were retrieved from NCBI according to their annotation. Redox-active Trx domains were manually extracted from this set. Abbreviations for QSOX correspond to species as listed in Figure S3. Branches corresponding to Trx domains of QSOX sequences are indicated with a pale-blue background. Viridiplantae and Metazoa are indicated with a green and slate-blue background, respectively. Plant and animal parasites are indicated with green and orange asterisks, respectively. **Figure S7.** Identity of an amino-acid residue in the vicinity of the CXXC motif of QSOX and PDI Trx domains distinguishes the two families. Representative motif sequences of QSOX Trx1 domains are shown on the left, with the serine/threonine residue common in metazoan QSOXs highlighted in green, and the histidine characteristic of plant QSOXs highlighted in gray. For comparison, all CXXC motifs from *H. sapiens* and *A. thaliana* QSOXs are shown on the right to demonstrate the near universality of proline, highlighted in yellow, at this position in PDI enzymes in both plant and metazoan species. Only a few QSOXs contain a proline. Click here for file
