# Supplementary material for: Diversification of Quiescin sulfhydryl oxidase in a preserved framework for redox relay
Source: BMC Evol Biol. 2013 Mar 19;13:70. doi: 10.1186/1471-2148-13-70 (PMC3616962; doi:10.1186/1471-2148-13-70)
Supplement: Additional file 2: Figure S1 — Taxonomic classification of species for which a QSOX sequence was identified. The given classification is based on the NCBI taxonomy browser. Figure S2. QSOX orthologs, paralogs, and splice variants in various taxonomic classifications. To emphasize the hierarchy between orthologs and paralogs, Neighbor-joining (NJ) trees were constructed for QSOX sequences of Alveolata, Nematoda, Stramenopiles, Viridiplantae, Arthropoda, and Chordata. Figure S3. Consensus maximum likelihood tree of QSOX sequences, protists excluded. In an attempt to improve scores at the root of major branches, QSOX sequences of protists were removed from the dataset. Figure S4. Consensus maximum likelihood tree of the Trx domain of QSOX sequences. The Trx domains of QSOX sequences were retrieved using the pattern detailed in the methods section. Abbreviations correspond to species as listed in Figure S3. Figure S5. Consensus maximum likelihood tree of the Erv domain of QSOX sequences. The Erv domains of QSOX sequences were retrieved using the pattern detailed in the methods section. Abbreviations correspond to species as listed in Figure S3. Figure S6. Example of a neighbor-joining tree of the Trx domain/s of QSOX and PDI sequences. PDI sequences were retrieved from NCBI according to their annotation. Redox-active Trx domains were manually extracted from this set. Abbreviations for QSOX correspond to species as listed in Figure S3. Branches corresponding to Trx domains of QSOX sequences are indicated with a pale-blue background. Viridiplantae and Metazoa are indicated with a green and slate-blue background, respectively. Plant and animal parasites are indicated with green and orange asterisks, respectively. Figure S7. Identity of an amino-acid residue in the vicinity of the CXXC motif of QSOX and PDI Trx domains distinguishes the two families. Representative motif sequences of QSOX Trx1 domains are shown on the left, with the serine/threonine residue common in metazoan QSOXs highlighted in green, [file 1471-2148-13-70-S2.docx]

**Supplementary Material**


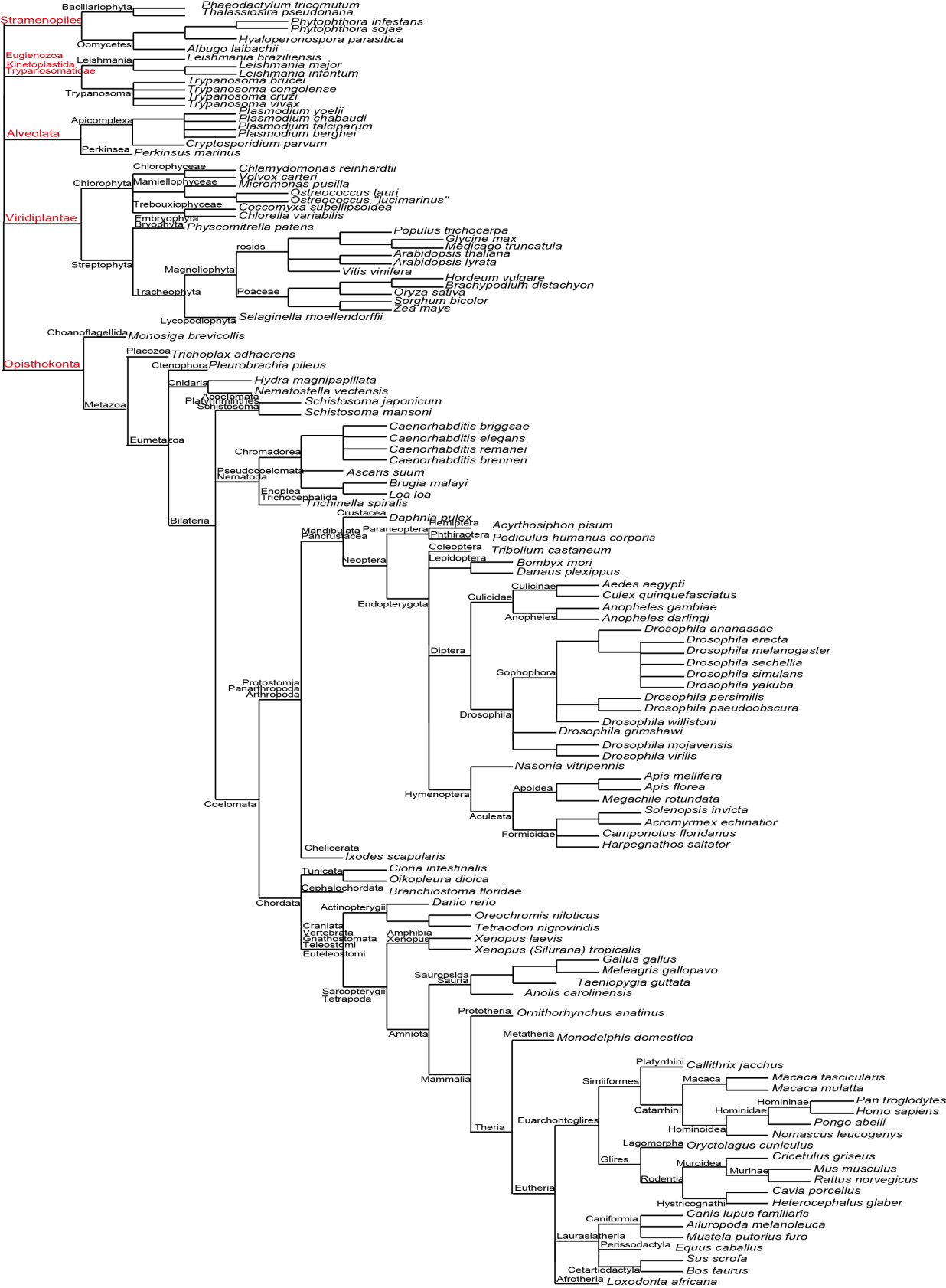


**Figure S1** **Taxonomic classification of species for which a QSOX sequence was identified.** The given classification is based on the NCBI taxonomy browser.


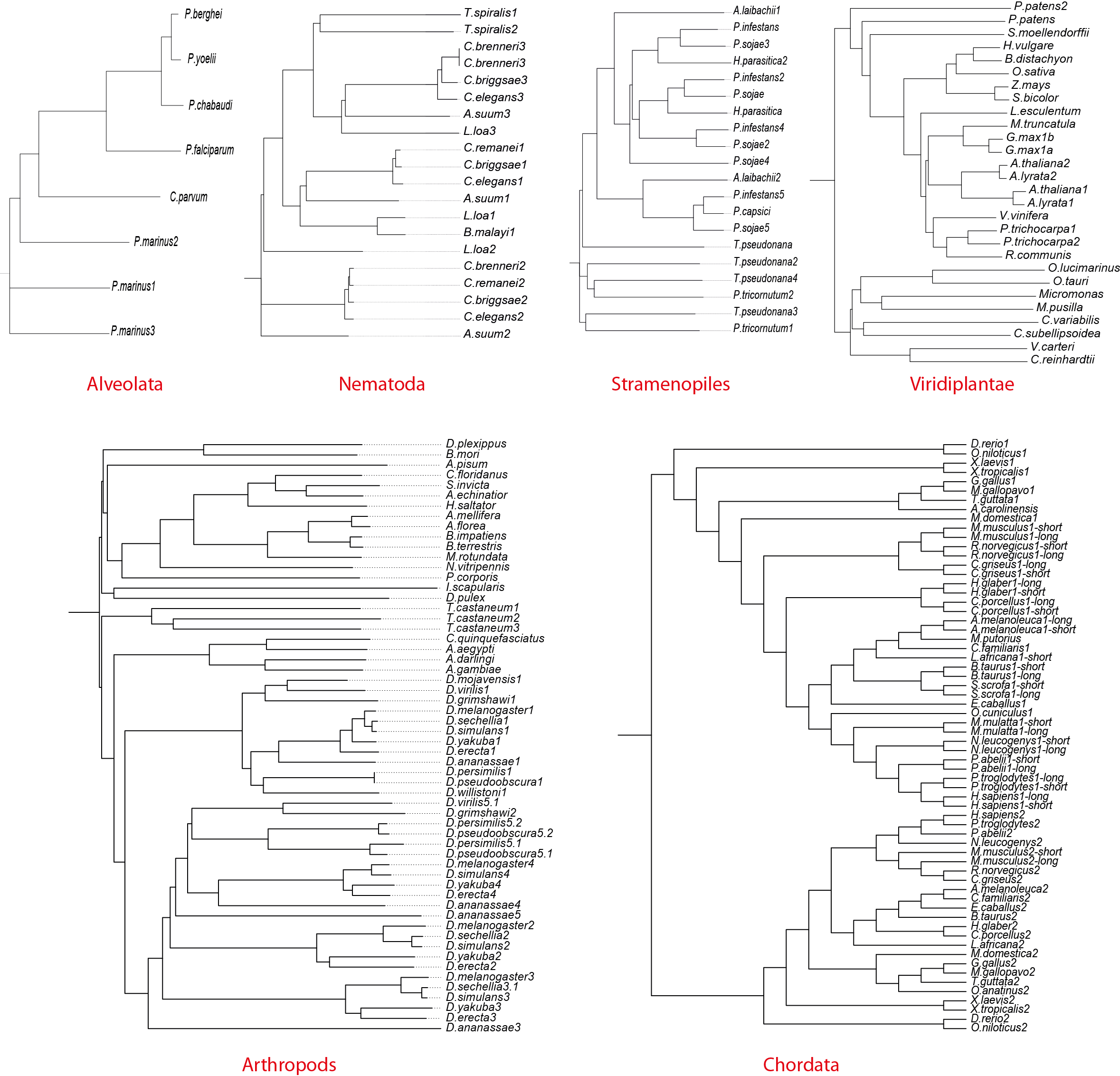


**Figure S2** **QSOX orthologs, paralogs, and splice variants in various taxonomic classifications.** To emphasize the hierarchy between orthologs and paralogs, Neighbor-joining (NJ) trees were constructed for QSOX sequences of Alveolata, Nematoda, Stramenopiles, Viridiplantae, Arthropoda, and Chordata.


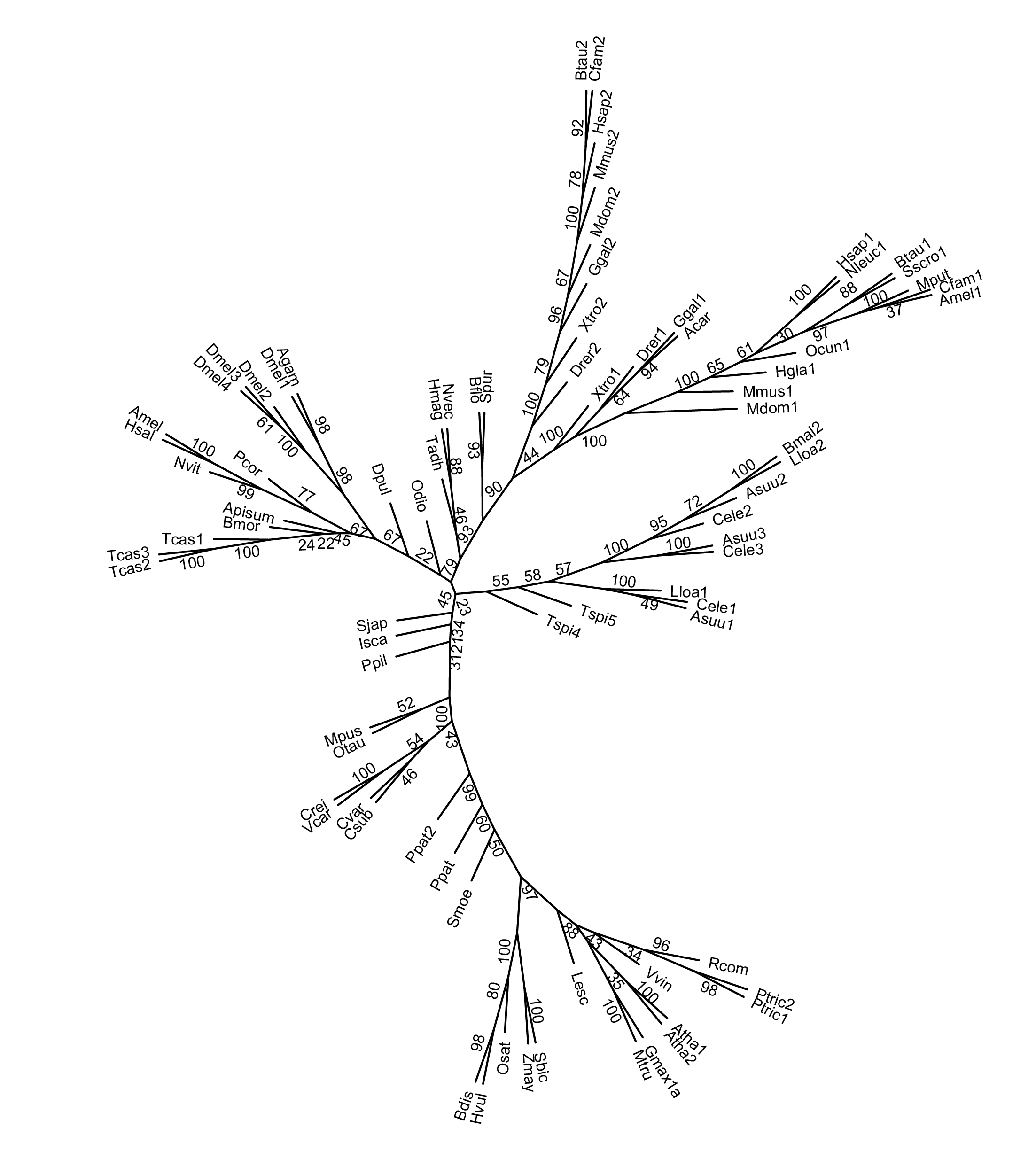


**Figure S3** **Consensus** **maximum likelihood tree of QSOX sequences, protists excluded.** In an attempt to improve scores at the root of major branches, QSOX sequences of protists were removed from the dataset. Sbic: *S. bicolor*, Zmay: *Z. mays*, Hvul: *H. vulgare*, Bdis: *B. distachyon*, Gmax: *G. max*, Mtru: *M*. *truncatula*, Ptric: *P. trichocarpa*, Rcom: *R. communis*, Vvin: *V. vinifera*, Lesc: *L. esculentum*, Atha: *A. thaliana*, Smoe: *S. moellendorffii*, Ppat: *P. patens*, Csub: *C. subellipsoidea*, Cvar: *C*. *variabilis*, Crei: *C. reinhardtii*, Vcar: *V. carteri*, Otau: *O. tauri*, Mpus: *M. pusilla*, Ptri: *P. tricornutum*, Tpse: *T. pseudonana*, Pinf: *P. infestans*, Pmar: *P. marinus*, Alai: *A. laibachii*, Cele: *C. elegans*, Asuu: *A. suum*, Lloa: *L. loa*, Tspi: *T. spiralis*, Ppil: *P. pileus*, Isca: *I. scapularis*, Sjao: *S. japonicum*, Cpar: *C. par*, Tbru: *T. brucei*, Linf: *L. infantum*, Pber: *P. berghei*, Odio: *O. dioca*, Dpul: *D. pulex*, Tcas: *T. castaneum*, Apis: *A. pisum*, Bmor: *B. mori*, Dmel: *D. melanogaster*, Agam: *A. gambiae*, Nvit: *N. vitripennis*, Amel: *A. mellifera*, Hsal, *H. saltator*, Pcor: *P. corporis*, Nvec: *N. vectensis*, Hmag: *H. magnipapillata*, Tadh: *T. adhaerens*, Spur: *S. purpuratus*, Bflo:*B*. *floridae*, Drer: *D. rerio*, Xtro: *X. tropicalis*, Ggal: *G. gallus*, Mdom: *M. domestica*, Mmus: *M*. *musculus*, Hsap: *H. sapiens*, Btau: *B. Taurus*, Cfam: *C. familiaris*, Acar: *A. carolinensis*, Hgla: *H.* *glaber*, Ocun: *O. cuniculus*, Nleu: *N. leucogenys*, Sscr: *S. scrofa*, Mput: *M. putorius*, Amel: *A*. *melanoleuca*.


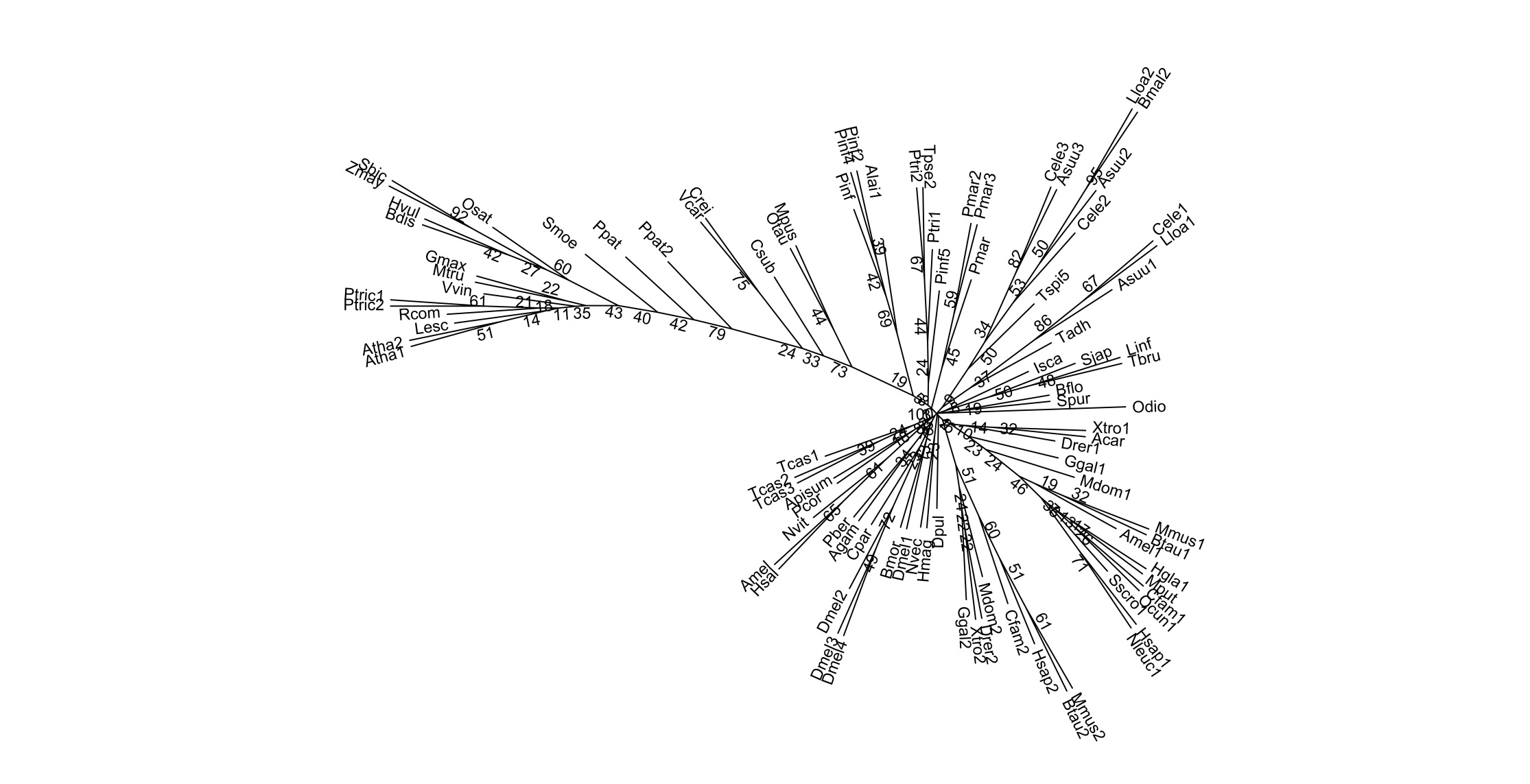


**Figure S4** **Consensus** **maximum likelihood tree of the Trx domain of QSOX sequences.** The Trx domains of QSOX sequences were retrieved using the pattern detailed in the methods section. Abbreviations correspond to species as listed in Figure S3.


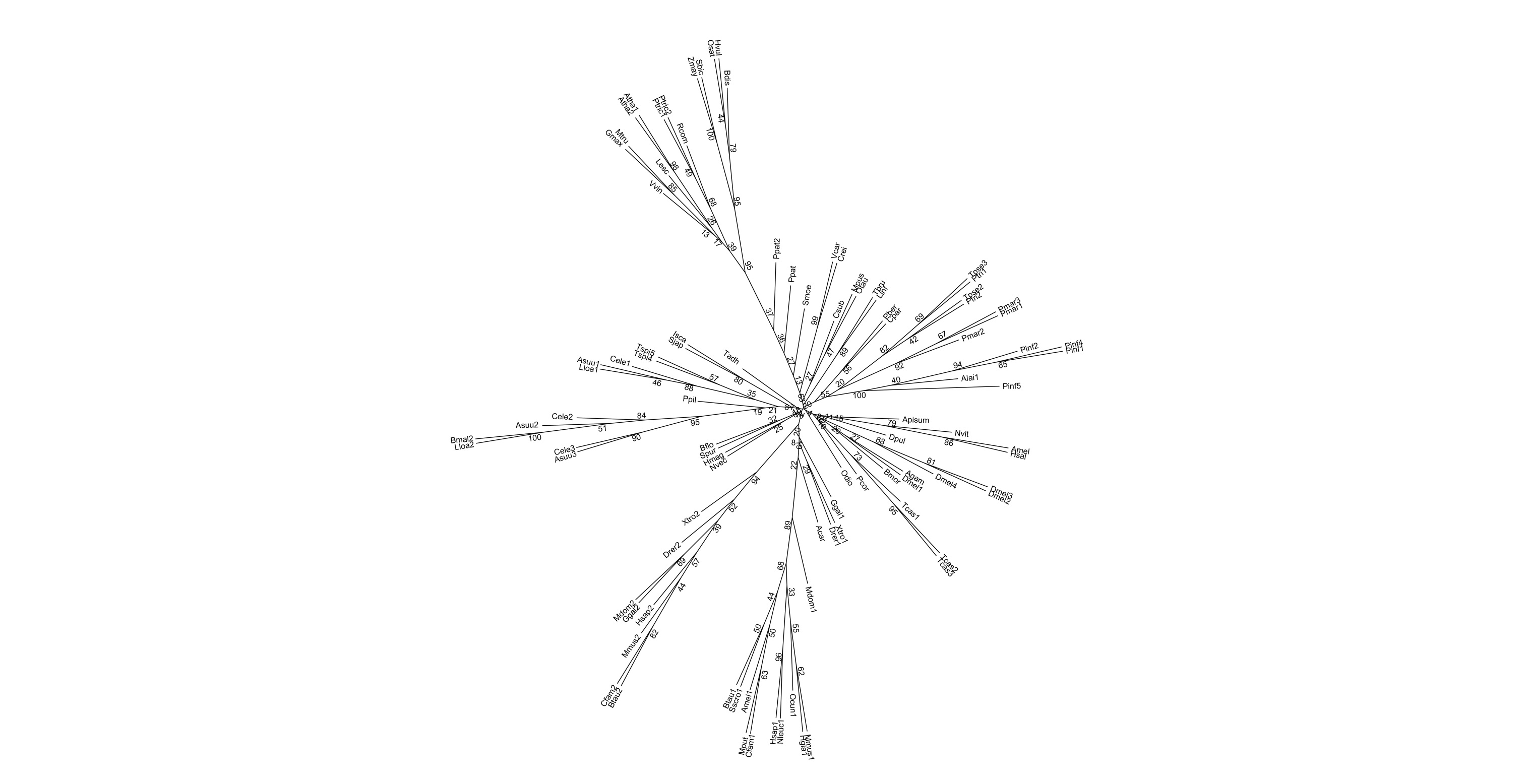


**Figure S5** **Consensus** **maximum likelihood tree of the Erv domain of QSOX sequences.** The Erv domains of QSOX sequences were retrieved using the pattern detailed in the methods section. Abbreviations correspond to species as listed in Figure S3.


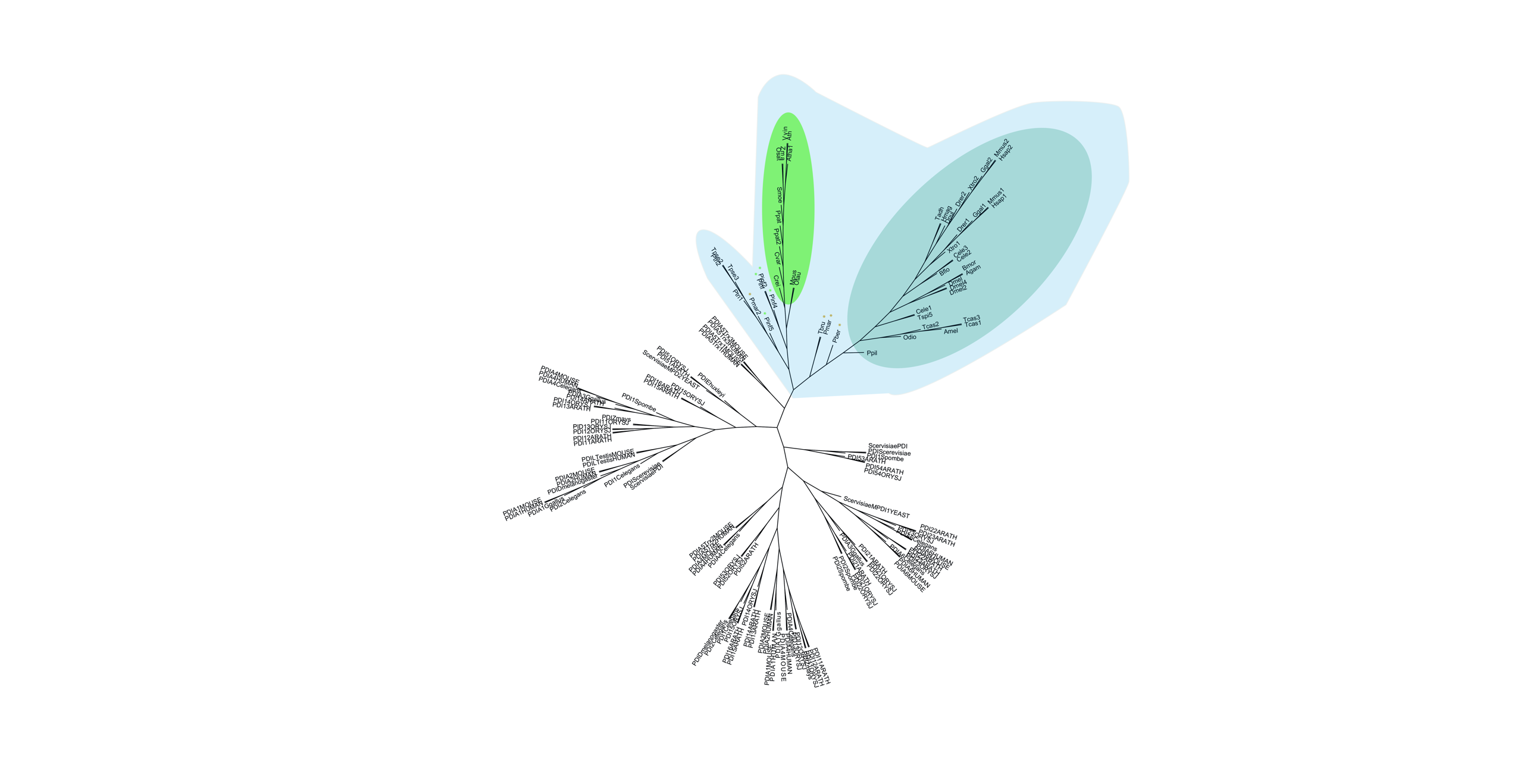


**Figure S6** **Example of a** **neighbor-joining tree of the Trx domain/s of QSOX and PDI sequences.** PDI sequences were retrieved from NCBI according to their annotation. Redox-active Trx domains were manually extracted from this set. Abbreviations for QSOX correspond to species as listed in FigureS3. Branches corresponding to Trx domains of QSOX sequences are indicated with a pale-blue background. Viridiplantae and Metazoa are indicated with a green and slate-blue background, respectively. Plant and animal parasites are indicated with green and orange asterisks, respectively.

**QSOX representatives**

*H. sapiens* SWCGHC

*C. familiaris* SWCGHC

*M. domestica* SWCGHC

*G. gallus* SWCGHC

*X. laevis* SWCGHC

*D. rerio* TWCGHC

*D. melanogaster* TYCGHC

*C. elegans* DWCGHC

*C. intestinalis* EWCGHC

*T. adhaerans* SWCGHC

*M. brevicollis* SWCGGC

*T. brucei* DGCGAC

*L. major*  DTCGHC

*A. thaliana* HWCPAC

*G. max* HWCPAC

*O. sativa* HWCPAC

*Z. mays* HWCPAC

*P. patens* HWCGAC

*M. pusilla* PWCPHC

*O. tauri*  PWCPAC

*P. infestans* PWCPHC

***H. sapiens* PDIs**

PDI PWCGHC

PWCGHC

ERp57 PWCGHC

PWCGHC

ERp72 PWCGHC

PWCGHC

PWCGHC

PDIP PWCGHC

PWCTHC

ERp18 SWCGAC

ERp46 PWCGHC

PWCGHC

PWCGHC

P5 PWCGHC

PWCGHC

PDIr PWCPPC

PWCGHC

PWCPHC

ERdj5 PGCSHC

PWCHPC

PWCGPC

TMX PWCPAC

TMX3 PWCGHC

TMX4 PWCPSC

***A. thaliana* PDIs**

At1g21750 PWCGHC

PWCGHC

At1g77510 PWCGHC

PWCGHC

At3g54960 PWCGAC

PWCGHC

At5g60640 PWCGHC

PWCGHC

At1g52260 PWCARS

PWCINC

At2g47470 PWCGHC

PWCGHC

At1g04980 PWCGHC

PWCGHC

At2g32920 PWCGHC

PWCGHC

At1g35620 PWCGHC

At3g20560 PWCYWS

At4g27080 PWCYWC

At4g27080 PWCYWC

At1g07960 PWCKHC

**Figure S7** **Identity of an amino-acid residue in the vicinity of the CXXC motif of QSOX and PDI Trx domains distinguishes the two families.** Representative motif sequences of QSOX Trx1 domains are shown on the left, with the serine/threonine residue common in metazoan QSOXs highlighted in green, and the histidine characteristic of plant QSOXs highlighted in gray. For comparison, all CXXC motifs from *H. sapiens* and *A. thaliana* QSOXs are shown on the right to demonstrate the near universality of proline, highlighted in yellow, at this position in PDI enzymes in both plant and metazoan species. Only a few QSOXs contain a proline.
